# The Cloning and Characterization of the *Enolase2* Gene of *Gekko japonicus* and Its Polyclonal Antibody Preparation

**DOI:** 10.3390/ijms14058787

**Published:** 2013-04-24

**Authors:** Jing Li, Ronghua Wu, Haijiao Chen, Youlang Zhou, Yan Li, Yongjun Wang, Yan Liu, Mei Liu

**Affiliations:** 1 Jiangsu Key Laboratory of Neuroregeneration, Nantong University, Nantong 226001, Jiangsu, China; E-Mails: lijing009@126.com (J.L.); wuronghua5@126.com (R.W.); 10510004@yjs.ntu.edu.cn (H.C.); youlangzhou@163.com (Y.Z.); hesaidan@126.com (Y.L.); wyjbs@yahoo.com.cn (Y.W.); 2 Clinical Laboratory, the Central Hospital of Huzhou, Huzhou 313000, Zhejiang, China

**Keywords:** *Gekko japonicus*, Molecular cloning, *Neuron specific enolase* (*NSE*), polyclonal antibody

## Abstract

The *enolase2* gene is usually expressed in mature neurons and also named neuron specific enolase (*NSE*). In the present study, we first obtained the *NSE* gene cDNA sequence by using the RACE method based on the expressed sequence tag (EST) fragment from the cDNA library of *Gekko japonicus* and identified one transcript of about 2.2 kb in central nervous system of *Gekko japonicus* by Northern blotting. The open reading frame of *NSE* is 1305 bp, which encodes a 435 amino-acid protein. We further investigated the multi-tissue expression pattern of *NSE* by RT-PCR and found that the expression of *NSE* mRNA was very high in brain, spinal cord and low in heart, while it was not detectable in other tissues. The real-time quantitative PCR was used to investigate the time-dependent change in the expression of the *NSE* mRNA level after gecko spinal cord transection and found it significantly increased at one day, reaching its highest level three days post-injury and then decreasing at the seventh day of the experiment. The recombinant plasmid of pET-32a-NSE was constructed and induced to express His fused NSE protein. The purified NSE protein was used to immunize rabbits to generate polyclonal antisera. The titer of the antiserum was more than 1:65536 determined by ELISA. Western blotting showed that the prepared antibody could specifically recognize the recombinant and endogenous NSE protein. The result of immunohistochemistry revealed that positive signals were present in neurons of the brain and the spinal cord. This study provided the tools of cDNA and polyclonal antibody for studying NSE function in *Gekko japonicus*.

## 1. Introduction

We know that adult mammalian spinal cord fails to regenerate after injury, but there are significant examples in which injured spinal cord can regenerate following some specific types of lesions in lots of lower vertebrates, such as in fish, urodele amphibians and lizards [1–3], or even in embryonic mammals [4]. Although the reason for the loss of the regeneration potential during the process of systematic evolution and individual development is poorly understood, it is still of great interest to demonstrate the intrinsic regeneration capacity [5–7]. Exploring the mechanisms of spinal cord regeneration in those lower vertebrate may result in new ideas to supply the strategy for human spinal cord injury.

In our laboratory, *Gekko japonicus* is used as an animal model of regeneration research, since the geckos are well known for their remarkable ability to regenerate its tail and get full movement and functional recovery in a certain time after amputation [3,8–10]. The gecko tail has major axial structure, including the spinal cord. Therefore, the understanding of tail regeneration in gecko could contribute to the molecular mechanism of mammalian spinal cord regeneration. To date, we have constructed a cDNA library of the brain and the spinal cord from *Gekko japonicus* [11], and the full length cDNAs of some interesting genes related to tail regeneration, such as PDGF-C, CD59, Brp44l [12–14], have been identified. We also explored the methods of neural cells isolation and culture and obtained two gecko immortal neural cell lines [15]. For the purpose of investigating the central nervous system of geckos, especially addressing diverse changes of injured neural cells after gecko CNS trauma, some species-specific neural cells marker genes need to be obtained. To date, the complete open read frame (ORF) information of the *GFAP* [16], *TUBB3* [17] gene and their corresponding polyclonal antibodies have been obtained in our laboratory. In the present study, we aimed to clone and characterize the *enolase2* gene.

Enolase, which catalyzes the interconversion of 2-phosphoglycerate to phosphoenolpyruvate, is a rate-limited enzyme in the glycolytic pathway. Mammalian enolase is composed of three isozyme subunits, α, β and γ, which can form homodimers or heterodimers, which are cell-type and development-specific [18]. Therein, the *enolase2* gene, which encodes one of the enolase isoenzymes, γ, is named neuron specific enolase (*NSE*), and the NSE protein was purified and investigated structurally, immunologically and functionally from rat whole brain extract in 1978 [19]; its cDNA was first identified by Sakimura K, *et al*. [20]. The enolase exists in three molecular forms in human brain: non-neuronal enolase (NNE; αα-enolase), neuron specific enolase (NSE; γγ-enolase) and the short-lived heterodimer αγ-enolase [21,22]. The NSE is a highly acidic homodimeric protein of 78 kDa, which has been found in the nervous system and also in many neuroendocrine cells and neuron derivative tumors [23–25]. From then on, *NSE* is usually viewed as the neuron and neural system developmental marker [26]. It is well known that traumatic brain or spinal cord injury or neuron-derived tumor usually results in neurons damage and NSE leaks into cerebrospinal fluid (CSF) or blood circulation system. In lots of recent studies, NSE was already widely used as a diagnostic biomarker of spinal cord trauma, brain injury and neuroendocrine tumors in humans [27–31]. The measurement of NSE protein levels in both serum and CSF following cerebral ischemia and traumatic head injury provides a reliable laboratory indicator of the degree of brain cell damage and may allow early prediction of prognostic outcome [32,33].

In this study, we cloned the full-length *NSE* cDNA sequence by RACE method based on an EST sequence from the cDNA library of the brain and the spinal cord of *Gekko japonicus* and then analyzed its homology with other species. Furthermore, we investigated some basic characteristics of *NSE* gene and prepared the rabbit polyclonal antibody of geckos NSE protein. To our knowledge, it is the first report of gecko *NSE* gene.

## 2. Results and Discussion

### 2.1. Molecular Cloning and Bioinformatic Analysis of *NSE*

**Figure 1:**
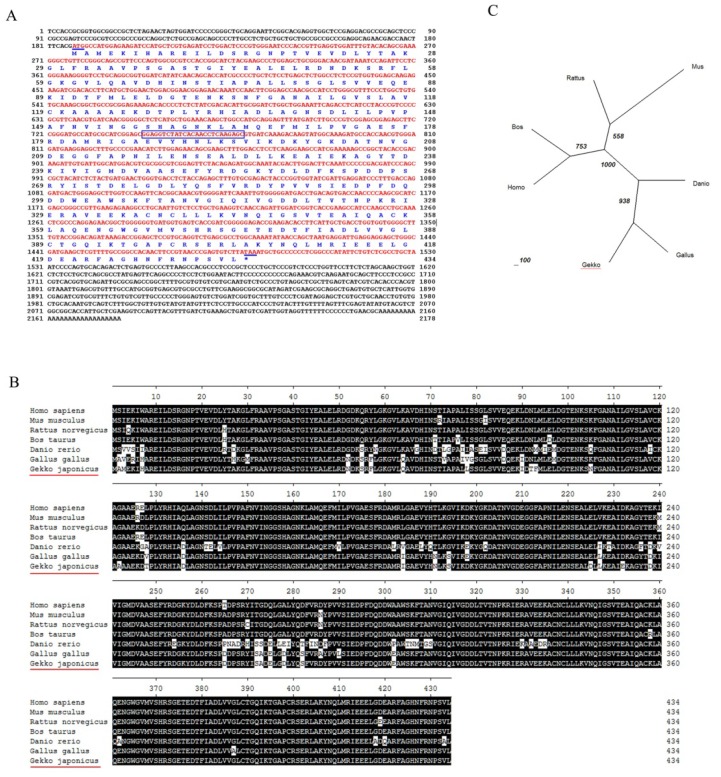
Molecular cloning and bioinformatic analysis of *NSE*. (**A**) The *NSE* cDNA (GenBank accession No. JQ080314) and its deduced amino acid sequence. The full-length cDNA of *NSE* was 1305 bp and the open reading frame encoded a polypeptide of 434 amino acids. The primers of RACE are boxed. The numbering of the nucleotide and amino acid sequences is shown on the right; (**B**) alignment of *NSE* using the MegAlign program (DNASTAR) by the CLUSTAL method. Shaded (with solid black) residues are the amino acids that match the consensus. *NSE* amino acid sequences were obtained from previously reported sequences in GenBank, including *Danio rerio*, *Bos taurus*, *Gallus gallus*, *Mus musculus*, *Rattus norvegicus* and *Homo sapiens*; (**C**) phylogenetic tree analysis of *NSE* gene from *Gekko japonicus* and other species was constructed by neighbor-joining methods within the package PHYLIP 3.5c. Bootstrap majority consensus values on 1000 replicates are indicated at each branch.

We cloned gecko *NSE* (Accession Number: JQ080314) cDNA based on the EST sequence from the brain and the spinal cord cDNA library of *Gekko japonicus*. The sequence from the RACE product and original EST fragment were joined to obtain a cDNA of 2178 bp, as shown in Figure 1A, and the longest open reading frame was 1305 bp, which encoded a polypeptide of 434 amino acids. The deduced amino acid sequence of NSE was analyzed by ProtParam tool in ExPASy, and the predicted molecular weight was 47.3 kDa, with the theoretical pI of 4.74. Based on the sequence comparison through a database search using BLAST program (http://www.ncbi.nlm.nih.gov) [34], the predicted gecko NSE protein revealed that it contains a conserved core pattern of Enolase (EC4.2.1.11) between 340 to 353 aa, which is similar to the known NSE protein of other species. The NSE amino acid sequences of different species, including *Danio rerio* (NP_001003848.1), *Bos taurus* (NP_001094595.1), *Gallus gallus* (NP_990207.1), *Mus musculus* (NP_038537.1), *Rattus norvegicus* (NP_647541.1) and *Homo sapiens* (NP_001966.1), were aligned using the MegAlign program of DNASTAR by the CLUSTAL method. The results showed that at the amino acid level, the similarity was 83%–98% with the previously reported sequences of *NSE* in NCBI (Figure 1B). The phylogenetic analysis of *NSE* gene from *Gekko japonicus* and other species showed that *Gallus gallus* has the closest relationship with *Gekko japonicus* among the species selected for analysis (Figure 1C).

### 2.2. *NSE* mRNA Transcript in CNS, the Expression Pattern in Different Tissues and mRNA Expression after Spinal Cord Transection in *Geckos*

**Figure 2:**
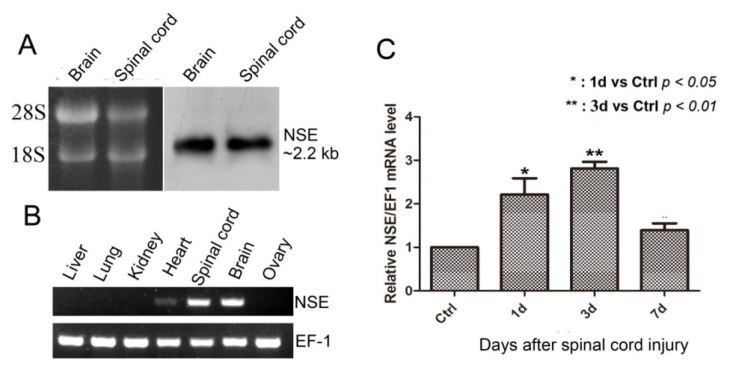
*NSE* mRNA transcript in CNS, the expression pattern in different tissues and mRNA expression after spinal cord transection in geckos. (**A**) The representative result of Northern blotting analyses of *NSE* mRNA in CNS of adult gecko. Ten micrograms of total RNA samples from brain and spinal cord of gecko were separated in 1% formaldehyde-denatured (*w*/*v*) agarose gel (**Left panel**), with the 18S and 28S rRNAs of corresponding tissues shown on the left. The 680 bp from NSE-open read frame (ORF) sequence was obtained by the PCR method and cloned into the pGEM-T Easy vector. Dig-labeled NSE riboprobes were synthesized *in vitro* from linearized plasmid above with T7 RNA polymerase. The hybridized bands were visualized with CDP-Star chemiluminescent substrate and recorded by X-ray film. The result of Northern blotting (**Right panel**) showed the length of *NSE* mRNA about 2.2 kb; (**B**) the representative results of semi-quantitative RT-PCR analyses of *NSE* mRNA in the different tissues of adult geckos, including liver, lung, kidney, heart, spinal cord, brain and ovary of adult geckos. The results revealed that the expression level of NSE mRNA was high in brain, spinal cord and low in heart, while it was not detectable in other tissues; (**C**) real-time qPCR analysis of NSE expression in the spinal cord after transection for the controls (Ctrl) and one, three and seven days post-injury. EF-1α was used for the quantitative normalization. * 1 d *vs*. Ctrl, *p* < 0.05; ** 3 d *vs*. Ctrl, *p* < 0.01.

We detected *NSE* mRNA transcript by Northern hybridization in brain and spinal cord from adult geckos. A positive band about 2.2 kb of *NSE* mRNA transcript was detected in the assay (Figure 2A), which was consistent with the cDNA of *NSE*, as described above. To investigate the expression pattern of *NSE* in different tissues of adult geckos, we performed semi-quantitative RT-PCR in liver, kidney, lung, heart, spinal cord, brain and ovary of adult geckos. The result revealed that the expression of *NSE* mRNA was very high in brain, spinal cord and low in heart, while it was not detectable in other tissues (Figure 2B). To investigate the time-dependent change in gecko *NSE* mRNA expression after spinal cord injury (SCI), we performed real-time quantitative PCR at 1, 3 and 7 d post-injury and found that the expression of *NSE* mRNA in the gecko spinal cord around the injury site increased from the second day, reaching its highest level three days after transection and then decreased by the seventh day of the experiment (Figure 2C).

### 2.3. The Titer of the Prepared Antiserum

**Figure 3:**
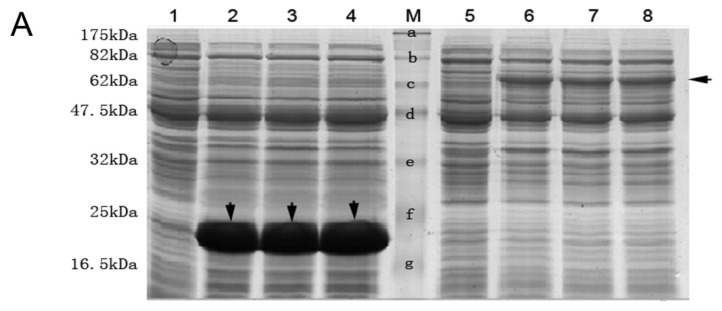

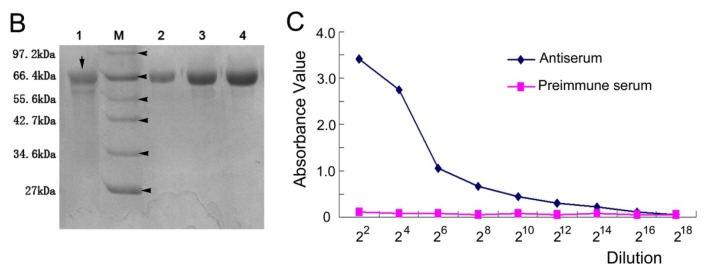
Expression, purification of His fusion *NSE* protein and the titer of the prepared antiserum. (**A**) Expression of His tagged NSE protein with isopropyl β-d-1-thiogalactopyranoside (IPTG) induction. Lane 1 and 5: the total protein of uninduced pET-32a vector and pET-32a-gNSE plasmid. Lane 2–4: the induced total protein of pET-32a vector with 0.1 mM, 0.5 mM and 1.0 mM IPTG, respectively. The bands showed His tag protein of pET-32a vector was about 17.5 kDa (↓) and the same expression efficiency at different IPTG concentration. Lane 6–8: the induced total protein of pET-32a-gNSE plasmid with 0.1 mM, 0.5 mM, 1.0 mM IPTG, respectively. The bands showed His fusion protein of pET-32a-gNSE plasmid was about 67 kDa (←) and the same expression efficiency at different IPTG concentration. Lane M showed the standard MW protein and their MW list on the left of the Lane 1, respectively; (**B**) Lane 1(↓) showed the result of purification of His fused gecko NSE protein. The Lanes of 2 to 4 were the BSA, protein and their loading amounts were 1 μg, 2 μg and 3 μg, respectively. Lane M showed the standard MW protein and their MW list on the left of the Lane 1, respectively. Staining assay using 10% SDS-PAGE and Coomassie brilliant blue R-250 was applied to assess the induced expression and the result of purification of the recombinant fusion protein; (**C**) Titer of the prepared antiserum was determined by ELISA. The blue curve showed the absorbance values of antiserum at different dilutions and the purple of pre-immune serum at the same dilution gradient.

**Figure 4:**
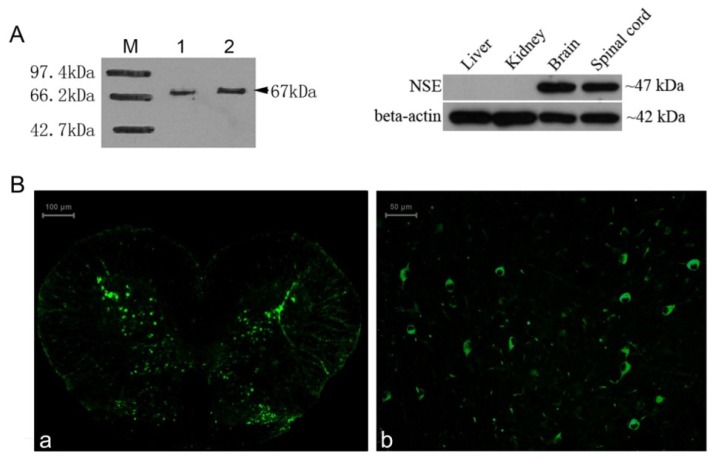
The representative results of Western blot and immunochemical analyses. (**A**) Lane 1 and 2 showed the result of Western blot by using the generated rabbit polyclonal antibody against His tagged gecko NSE protein and the commercial monoclonal antibody against His protein, both bands showed the MW of His-tagged gecko NSE protein was about 67 kDa. The right panel showed the result of Western blot by using the generated rabbit polyclonal antibody against NSE protein obtained from gecko liver, kidney, brain and spinal cord, and the bands were about 47 kDa. The beta-actin was used as the internal control; (**B**) The immunochemical assay showed specificity of the polyclonal antibody on the gecko spinal cord (**a**) and brain (**b**) sections. NSE was expressed in cytoplasm of mature neurons, and the fluorescence signals were mainly distributed in grey matter of spinal cord and extensively detected in brain.

The recombinant plasmid pET-32a-gNSE was constructed and induced to express the fusion protein by isopropyl β-d-1-thiogalactopyranoside (IPTG) under the different concentration of 0.1, 0.5 and 1.0 mM (Figure 3A). The fusion NSE protein was purified and electrophoresed by 10% SDS-PAGE (Figure 3B) and further confirmed by Western blotting using anti-His monoclonal antibody and anti-NSE antiserum (Figure 4A, left panel). The concentration of purified fusion protein was 11.29 mg/mL quantified by the BCA method. Then, the concentrated fusion protein was used to immunize rabbits to generate the anti-gNSE rabbit serum. The titer of the antiserum was more than 1:65536 (2^16^), determined by ELISA (Figure 3C).

### 2.4. Identification of the *NSE* Polyclonal Antibody by Western Blot and Immunochemistry Analysis

The result of Western blotting showed a band at about 47 kDa (Figure 4A, lane 3,4) from total proteins of adult gecko brain and spinal cord using NSE antibody at dilutions 1:1000. The result of immunohistochemistry showed that the neurons in the sections of normal gecko spinal cord and brain were clearly labeled with the anti-gNSE polyclonal antibody (Figure 4Ba,b) and the positive signal of NSE mainly distributed in grey matter of spinal cord and extensively detected in brain.

## 3. Materials and Methods

### 3.1. Animals and Spinal Cord Injury

Adult *Gekko japonicus* were from the laboratory animal center of Nantong University. They were housed in an air-conditioned room with a controlled temperature of 26 °C and saturated humidity and freely fed mealworms and given water during the whole experiment. All experimental protocols applied to animals were given prior approval by the Laboratory Animal Care and Use Committee of the medical school. We conducted cooling anesthesia to minimize their suffering. Geckos received complete spinal cord transections at the L10–11 lumbar vertebrae level using procedures in our lab described previously [16]. Briefly, after laminectomy, the spinal cord was transected using iridectomy scissors. The severed ends of the cord typically retracted 1–2 mm and were inspected under a surgical microscope to ensure complete transection. For the sham-injury controls, the geckos underwent the laminectomy without transection. Then, the muscles and skin were closed. Followed by placing geckos in a temperature- and humidity-controlled chamber overnight, the animals were returned to their cages and allowed to survive for 1, 3 or 7 days before being sacrificed.

### 3.2. Molecular Cloning of Full-Length cDNA of *NSE*

The EST sequence, which is homologous to chicken enolase gene (Accession: NP990207), was obtained from the cDNA library of the gecko brain and spinal cord. To obtain the full-length transcript of enolase gene, the rapid amplification of cDNA ends (RACE) reaction was performed using a commercially available kit (Clontech, Mountain View, CA, USA). Total RNA was extracted from the brain and spinal cord of the gecko using Trizol reagent (Invitrogen) and purified by pheno-chloroform extraction again after DNase I digestion. The primers designed for RACE amplification were following: gene-specific primer (GSP1); 5′-gga ggt cta tca caa cct caa gag c-3′; nested gene specific primers (NGSP); forward: 5′-caa gga agc cat cga aaa ag-3′; reverse: 5′-cag ctc cca gtc atc tgt ca-3′. The procedures of RACE and PCR were performed according to the kit instructions. The product(s) of PCR were gel-purified and then sequenced. The longest cDNA sequence was obtained by combining fragments with overlapped region. All the primers synthesis and DNA sequencing in this study were serviced by Shanghai Invitrogen Biotechnology Co., Ltd. (Shanghai, China).

### 3.3. Northern Blot Analysis

The PCR product of 680 bp from NSE-ORF sequence was obtained using NSE-probe primer-Forward 5′-gga acg gag aac aaa tcc aa-3′ and NSE-probe primer-Reverse 5′-ggg ttg gtc act gtc agg tc-3′ and identified by DNA sequencing and then was cloned into pGEM-T Easy vector (Promega, Madison, WI, USA). Digoxigenin (Dig)-labeled *NSE* riboprobes were synthesized *in vitro* from linearized plasmid above with T7 RNA polymerase, following the DIG-UTP supplier’s instructions (Roche, Mannheim, Gemany). Ten micrograms of total RNA samples prepared with Trizol reagent, respectively, from brain and spinal cord of gecko were separated in 1% formaldehyde-denatured (*w*/*v*) agarose gel and transferred to nylon membrane. The procedures of Northern blot followed the instructions, as described in our previous studies [12]. The hybridized bands were visualized with CDP-Star chemiluminescent substrate (Roche, Mannheim, Gemany) and recorded by X-ray film.

### 3.4. RT-PCR and Real-Time Quantitative PCR

The primers for RT-PCR were designed by Primer Premier 5.0 software and described as below. NSE-forward: 5′-agg ctt tgc ccc gaa cat-3′; NSE-reverse: 5′-cca cca cca ggt cag caa t-3′; EF-1α-forward: 5′-cat gtc gat tct ggc aag tc-3′; EF-1α-reverse: 5′-ctg gct gta agg tgg ctc ag-3′. The total RNA of geckos liver, lung, kidney, heart, spinal cord, brain and ovary were prepared. The first strand cDNA was synthesized using Omniscript Reverse Transcription Kit (QIAGEN, Valencia, CA, USA) in a 20 μL reaction system containing 2 μg total RNA. Then, 1 μL aliquot from the first-strand cDNA synthesized was amplified with primers designed to investigate the expression of *NSE* in the 25 μL of PCR amplification reaction. Normalization was carried out at the same time by amplification of EF-1α (eukaryotic elongation factor 1 alpha) under the same condition described above. Meanwhile, a negative control without the first-strand cDNA was also performed. Gels were stained with GelRed™ (Biotium, Hayward, CA, USA) and visualized using a GeneGenius imaging system (SynGene, Frederick MD, USA). The identity of the RT-PCR products was confirmed by DNA sequencing.

The primers of real-time PCR were follows: *NSE* sense 5′-gct gcg gga caa cga taa-3′ and antisense 5′-gcg ttg gct ccg aag ttg-3′; *EF-1α* sense 5′-ctg gct gga atg gag ata aca-3′ and antisense 5′-gaa gag gaa gac gca gag gtt-3′. Total RNA and reverse transcription were performed as above from the 1 cm section of the spinal cord, including 0.5 cm above and below the injury site centered around L10–11 for the control and injured animals (*n* = 6 each) 1, 3 and 7 days after SCI. The reaction mixtures included 10 μL of 2× Fast Evagreen qRCR Master Mix (Biotium, Hayward, CA, USA), 2 μL of 10× ROX (Biotium, Hayward, CA, USA), primers and 1 μL of cDNA. Real-time PCR was performed in a StepOne Real-time PCR system (ABI Applied Biosystems, Grand Island, NY, USA). The thermal cycling program consisted of 2 min at 96 °C, followed by 45 cycles of 15 s at 96 °C and 1 min at 60 °C. Data collection was done during the 60 °C extension step. To account for the variability in the total RNA input, the expression of *NSE* was normalized to *EF-1α*. In addition, a negative control without the first-strand cDNA was also performed. The relative expression was calculated using the comparative 2^−ΔCt^ method, and all data were expressed as the means ± SD. Differences between groups were analyzed by one-way analysis of variance (ANOVA) using SPSS software. Significance was considered at *p < 0.05 and **p < 0.01.

### 3.5. Bioinformatic Analysis of *NSE*

The sequence of *NSE* was analyzed for coding probability with the DNATools programs. Comparison against the GenBank protein database was performed using the BLAST network server (http://www.ncbi.nlm.nih.gov/BLAST) [34]. The theoretical molecular weight and pI of the NSE protein were analyzed by ProtParam tool in ExPASy (http://www.expasy.org) [35]. Multiple protein sequences were aligned using the CLUSTAL method in the DNASTAR software package. The phylogenetic tree was constructed by the neighbor-joining method within the PHYLIP 3.5c software package using 1000 bootstrap replicates [36].

### 3.6. Expression, Purification of His Fusion NSE Protein and Preparation of Anti NSE Polyclonal Antibody

The open reading frame of *NSE* was amplified by PCR, which was performed in accordance with the User Manual of Advantage^®^ 2 Polymerase Mix (Clontech, Mountain View, CA, USA). The primers used in the amplification reaction incorporating *Eco*RI and *Xho*I restriction enzyme sites were as follows. Forward primer: 5′-ccg *gaattc* atggccatggagaagatcc-3′; reverse primer: 5′-ccg *ctcgag* ttaagacactcgggttacg-3′. The amplification products were digested with *Eco*RI/*Xho*I and then ligated into pET-32a vector to construct a recombinant plasmid named pET-32a-NSE. Then, the plasmid was transformed into competent *E. coli* strain BL21 cells for the purpose of recombinant protein expression. Induction conditions for the expression of recombinant protein were optimized in terms of different temperatures, concentrations of IPTG and durations of induction for the purpose of maximum production. Un-induced control culture and the pET-32a vector control culture were analyzed in parallel. Ten-percent SDS-PAGE and Coomassie brilliant blue R-250 staining was applied to assess the expression level of the recombinant fusion protein. The purification of the His-tagged NSE protein was performed as Fakhri Haghi *et al*. [37] described with some modifications by affinity chromatography on a nickel-nitrilotriacetic acid (Ni-NTA) gel matrix (Qiagen, Valencia, CA, USA) under denaturing conditions following the manufacturer’s instruction. The concentration of eluted His tag NSE protein was determined by Pierce BCA Protein Assay Kit (Thermo, Rockford IL, USA), and the purity of the His-NSE protein was determined by SDS-PAGE and Coomassie blue staining.

New Zealand white rabbits (1.5–2 kg) were injected subcutaneously with the mixture of 600 μg purified fusion NSE protein and an equal volume of complete Freund’s adjuvant (Sigma, St. Louis, MO, USA). Two weeks later, the second and the third injections in different region were performed at a one-week interval in order to boost the immune reaction, both with the mixture of 200 μg NSE fusion protein and an equal volume of incomplete Freund’s adjuvant. Ten days after the last immunization, the antiserum was harvested from rabbits by cardiac puncture. Subsequently, the rabbit antiserum was precipitated with saturated ammonium sulfate to extract the IgG fraction, which was then analyzed by SDS-PAGE and stored at −80 °C for further use.

### 3.7. Titer Determination of Anti-NSE Antiserum by ELISA

We performed ELISA assay following the previous description [38] with modifications. The purified NSE fusion protein as antigens were diluted to 50 μg/mL and coated on 96-well plates (100 μL/well) at 4 °C overnight. After three washes with PBS-T, 200 μL BSA at 50 g/L was added, and the plate was incubated at room temperature for 30 min and another 2 h at 37 °C with the addition of 100 μL anti-NSE antiserum with different dilutions (from 1:2^2^ to 1:2^18^) and preimmune serum with the same dilution gradient. Then 100 μL HRP-conjugated goat anti-rabbit IgG with the dilution of 1:10,000 was added into the wells and incubated at 37 °C for 30 min after washing. Freshly prepared 3,3′,5,5′-tetramethylbenzidine (TMB) solution was used as substrate to detect peroxidase activity, and color development was stopped 15 min later by 2 M of H_2_SO_4_. The absorbance was measured at 450 nm in the plate reader.

### 3.8. Western Blotting

The fusion NSE protein purified from bacteria, and NSE proteins extracted from liver, kidney, brain and spinal cord were used as antigens to evaluate the reactivity of the polyclonal antibody. The beta-actin was used as an internal control of tissue proteins. The procedures of Western blotting were performed as described in our previous study [12]. Briefly, the blotted PVDF membrane was incubated with either our anti-NSE polyclonal antibody or anti-His tag antibody (Sigma, St. Louis, MO, USA) at 4 °C overnight and was immersed in horseradish peroxidase labeled goat anti-rabbit IgG with dilution of 1:10,000 for 1 h at room temperature at next day. Finally, the membrane was developed with diaminobenzidine substrate buffer, detected by ECL Western Blot Detection Reagents (Thermo, Rockford, IL, USA) and exposed to X-ray film.

### 3.9. Immunohistochemistry

Immunohistochemistry staining was applied to assess the specificity of the polyclonal antibody. About 10 μm thick frozen sections freshly prepared from brain and spinal cord from adult gecko were blocked by 10% goat serum and 0.3% Triton X-100 at 37 °C for 30 min followed by incubation with rabbit anti-NSE polyclonal antibody with the dilution of 1:200. After three washes with PBS, fluorescein isothiocyanate (FITC)-labeled goat anti-rabbit IgG was used to detect the immunological reaction. Images were captured by inverted fluorescence microscope (Leica, Wetzlar, Germany).

## 4. Conclusions

The spinal cord injury had been investigated extensively in many species from fish to human, while the reptile animal was absent among the popular model for spinal cord injury and regeneration. The geckos show obvious ability to regenerate spinal cord, both in spinal cord transection and tail amputation models in our previous work, and it could be a potential candidate species for regeneration research in reptiles. In this study, we first identified the gecko NSE cDNA, encoding a 435-amino acid protein. It has a high homology with that of other vertebrates. The phylogenetic analysis of the gecko *NSE* and that of other species showed that *Gallus gallus* has the closest relationship with *Gekko japonicus* among the species of *Danio rerio*, *Bos taurus*, *Gallus gallus*, *Mus musculus*, *Rattus norvegicus* and *Homo sapiens.* We further investigated the mRNA transcript in the gecko central nervous system (CNS) and identified just a single transcription product of about 2.2 kb in the sample from brain and spinal cord. The result of multi-tissue expression pattern by RT-PCR showed high mRNA level of NSE in brain and spinal cord, but it also considerable expression in adult gecko heart compared to the non-detectable expression in kidney, liver, lung and ovary. We also performed the real-time quantitative PCR to investigate the time-dependent change in gecko *NSE* mRNA expression after spinal cord transection, and the result showed that the expression of *NSE* mRNA significantly increased at one day, reaching its highest level three days after transection and then decreased by the seventh day of the experiment. Since the *NSE* is now generally used as a specific marker of neuron, we further prepared the anti-gecko *NSE* polyclonal antibody, and the prepared polyclonal antibody could specifically recognize the recombinant and endogenous *NSE* protein from brain and spinal cord of *Gekko japonicus*, both in the Western and immunohistochemistry assay. The raised antibody provides a potential tool to determine the cellular localization and expression level of *NSE* protein during spinal cord injury and regeneration, which could benefit explicating the biological significance of the NSE protein in regeneration research.
